# Effectiveness of Interspinous Process Devices in Managing Adjacent Segment Degeneration Following Lumbar Spinal Fusion: A Systematic Review and Meta-Analysis

**DOI:** 10.3390/jcm13175160

**Published:** 2024-08-30

**Authors:** Harris Mangal, David Felzensztein Recher, Roozbeh Shafafy, Eyal Itshayek

**Affiliations:** 1 Medway NHS Foundation Trust, Gillingham ME7 5NY, UK; h.mangal@nhs.net; 2 Rabin Medical Center, Petah Tikva 4941492, Israel; eyalitshayek@clalit.org.il; 3 Royal National Orthopaedic Hospital, Stanmore HA7 4LP, UK; roozbeh.shafafy@nhs.net

**Keywords:** interspinous device, adjacent segment disease, lumbar spinal fusion, meta-analysis, systematic review

## Abstract

**Background:** Adjacent segment degeneration (ASD) is a significant complication following lumbar spinal fusion, often necessitating further surgical interventions and impairing patient outcomes. Interspinous process devices were introduced as an alternative treatment for spinal stenosis and degenerative spondylolisthesis and can potentially reduce the incidence of ASDd. This systematic review and meta-analysis aims to evaluate the effectiveness of interspinous process devices or IPDs in managing ASD following a previous spinal fusion compared to traditional fusion techniques. **Methods:** Electronic databases, including PubMed, Embase, and the Cochrane Library, were queried for studies assessing IPDs against traditional lumbar fusion methods for managing ASD after previous lumbar fusion, which had been published between January 2014 and the present. Statistical analysis was conducted using Review Manager 5.4. **Results:** Seven retrospective cohort studies involving 546 patients met the inclusion criteria. The analysis revealed that IPDs were associated with a statistically significant reduction in the incidence of ASD (OR = 0.28, 95% CI: 0.16 to 0.51, *p* < 0.0001, and I^2^ = 0% after excluding outliers). The ODI demonstrated a non-significant trend towards improved outcomes with IPDs at the 2-year follow-up (SMD = −3.94; 95% CI: −11.72 to 3.85). Range of motion (ROM) was better preserved with IPDs compared to fusion (SMD = 0.00, 95% CI: −0.41 to 0.41, *p* = 1.00, I^2^ = 60%). The visual analogue scale or VAS lower back pain scores were significantly reduced at the 2-year follow-up (SMD = −0.69, 95% CI: −1.18 to −0.19, *p* = 0.006, and I^2^ = 74%). VAS leg pain showed consistent improvements (SMD = −0.29; 95% CI: −0.63 to 0.04). Intraoperative blood loss was significantly lower with IPDs (SMD = −2.07; 95% CI: −3.27 to −0.87, *p* = 0.0007, and I^2^ = 95%), and operation times were shorter (SMD = −2.22, 95% CI: −3.31 to −1.12, *p* < 0.0001, and I^2^ = 94%). **Conclusions:** The judicious use of IPDs might benefit a subset of patients, particularly those who are not suitable candidates for major corrective surgery.

## 1. Introduction

The adoption of advanced spinal instrumentation and imaging technologies has greatly facilitated lumbar arthrodesis procedures by improving success rates and increasing the number of fusion surgeries performed [1,2]. Despite this, adjacent segment degeneration (ASD) remains a significant challenge, frequently necessitating additional surgical interventions and more extended hospital stays, as well as impairing post-surgical flexibility. ASD involves the progressive degeneration of spinal segments above or below the fusion site, as suggested by radiographic findings rather than clinical symptoms, due to altered spinal biomechanics and increased mechanical stress on these segments [3,4,5]. These changes can culminate in symptomatic adjacent segment disease (ASDis), which manifests clinically with pain and functional impairment. The prevalence of asymptomatic ASD occurs in up to 84% of patients, while symptomatic ASDis occurs from 1% to 43%, following lumbar fusion [6,7,8,9].

Interspinous process devices (IPDs) have been introduced as a less invasive alternative to traditional fusion techniques, working in conjunction with inter-body fusion and/or lumbar decompression or as a standalone device, aiming to provide dynamic stabilisation and preserve motion at the treated segment [10,11]. Although contraindicated for pars fractures, high-grade spondylolisthesis, and severe osteoporosis, ISPs can still be used to treat myriad spinous diseases, including degenerative disc disease, low-grade spondylolisthesis, and lumbar spinal stenosis and/or lumbar spinal instability [11]. The theoretical advantage of IPDs lies in their ability to reduce mechanical stress on adjacent segments by maintaining mobility and distributing loads more evenly across the spine. However, despite the apparent rationale behind its use in spinal stenosis, the significance of its impact on degenerative disc disease has yet to be established [12]. Several mechanisms of action have been purported, such as restoring foraminal height, maintaining stability, and unloading the facet joints and posterior annulus by distraction [11,12,13,14,15]. This approach could potentially delay or prevent the onset of ASD. However, the clinical efficacy of IPDs in achieving these goals remains a subject of debate, with studies reporting mixed outcomes regarding their effectiveness in managing ASD [16,17,18,19].

The need for a comprehensive meta-analysis on the effectiveness of IPDs in managing ASD arises from several critical considerations. First, the growing utilisation of IPDs in clinical practice underscores the importance of understanding their impact on adjacent segment health. While IPDs are designed to mitigate the adverse effects of spinal fusion, the variability in study designs, patient populations, and outcome measures has led to inconsistent findings across the literature. Previous meta-analyses have primarily focused on general outcomes such as pain relief and functional improvement, often overlooking the specific issue of ASD [20,21]. This meta-analysis will be the first to critically evaluate the effectiveness of interspinous process devices, using the most up-to-date evidence, in managing adjacent segment degeneration.

Moreover, potential areas of concern include the long-term biomechanical effects of IPDs on spinal kinematics and load distribution, which may need to be fully understood or adequately addressed in individual studies. There is also a need to evaluate the safety profile of IPDs since complications such as device migration, infection, and local tissue reactions could impact their overall efficacy and patient acceptance. Additionally, understanding the comparative effectiveness of IPDs versus traditional gold-standard fusion techniques in preventing or delaying ASD is crucial for informing clinical decision-making and optimising patient outcomes.

The primary objective of this meta-analysis is to systematically assess the effectiveness of IPDs in managing ASD by synthesising data from multiple studies. This analysis will focus on specific goals, including:

- Comparative Effectiveness: Evaluating the outcomes of IPD implantation compared to traditional lumbar fusion and other fusion techniques, specifically regarding adjacent segment health and overall patient outcomes.
- Safety Profile: Analysing the incidence and types of adverse events associated with IPDs versus fusion surgeries, to determine the relative safety of these devices.
- Long-term Outcomes: Assessing the durability and long-term efficacy of IPDs in preventing or delaying the progression of ASD, given the potential for these devices to provide sustained benefits or, conversely, long-term complications.
- Biomechanical Impact: Investigating the biomechanical effects of IPDs on spinal kinematics and load distribution, including any changes in the adjacent segments’ motion and stress patterns.

## 2. Materials and Methods

**Figure 1:**
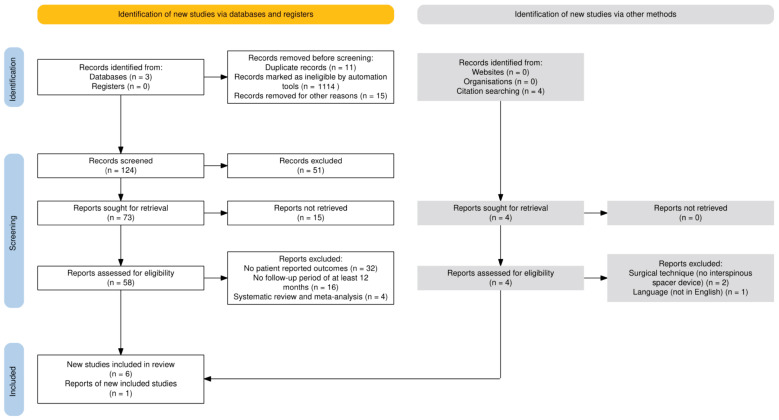
PRISMA flow diagram for the literature search on the effectiveness of interspinous spacer devices (IPDs) in managing adjacent segment degeneration (ASD) using the PRISMA flow diagram tool [23].

This meta-analysis used the preferred reporting items for systematic reviews and meta-analyses (PRISMA) statement [22] (Figure 1). The review was not registered in PROSPERO.

### 2.1. Search Strategy

A comprehensive literature search was conducted across multiple electronic databases, including PubMed, Embase, and the Cochrane Library, for studies published within the past decade. Randomised controlled trials, cohort, and comparative studies assessing the effectiveness of an interspinous spacer device compared to a control, and with a minimum follow-up period of 12 months, were selected. Additionally, the references of identified and relevant review articles were hand-searched to ensure comprehensive coverage. The search strategy included keywords and medical subject headings (MeSH) terms related to “interspinous spacer devices”, “adjacent segment degeneration and disease”, and “lumbar spine surgery”. A combination of Boolean operators was used to maximise search sensitivity, and the search was further refined using the patient, intervention, comparison, and outcome or PICO search strategy and was tailored for each database to meet the objectives of the meta-analysis. An example search strategy for PubMed was: (adjacent segment degeneration OR adjacent segment disease) AND (interspinous process device OR IPD) AND (“visual analogue scale” OR “VAS”) OR (“Oswestry Disability Index” OR “ODI”) OR (“biomechanical”) OR (“patient-reported outcome”) OR (“cost-effectiveness”) OR (“radiographic”).

### 2.2. Selection Criteria

**Figure 2:**
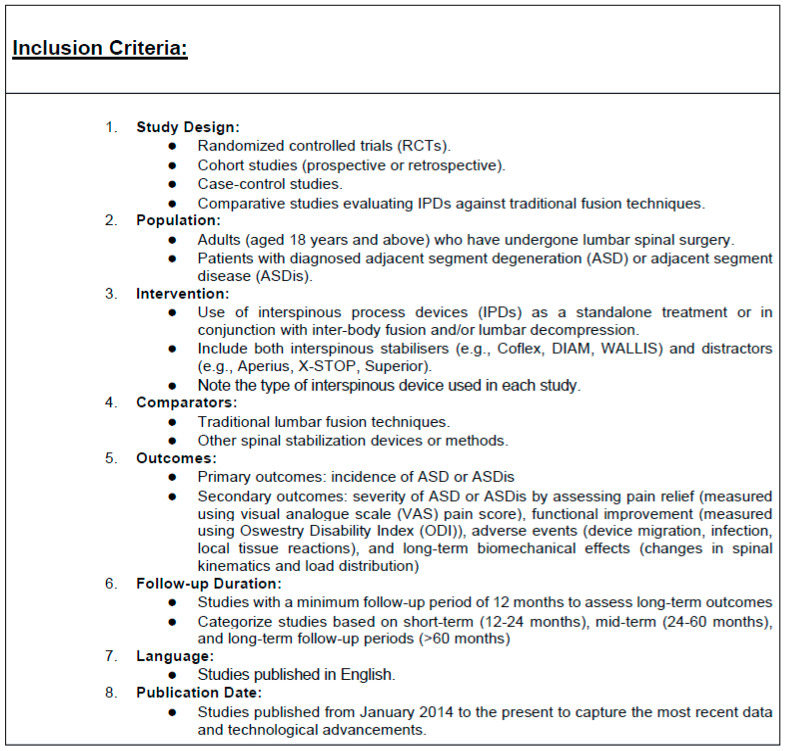
Inclusion criteria for the database search.

**Figure 3:**
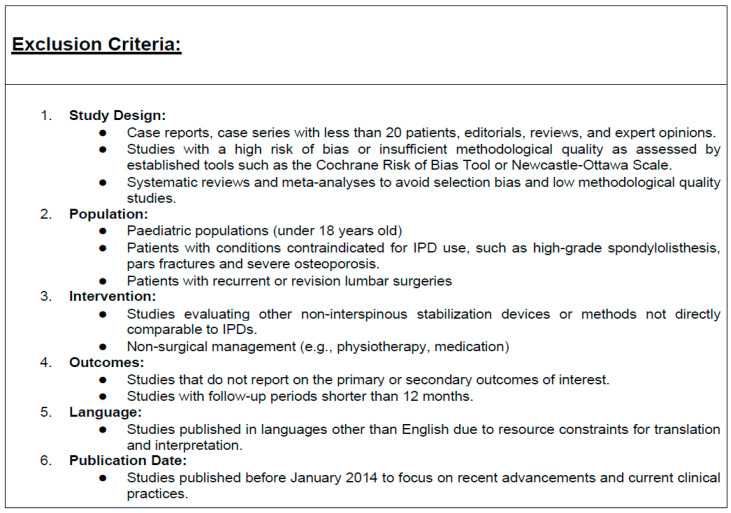
Exclusion criteria for the database search.

Rigorous selection criteria, with a detailed breakdown of the inclusion and exclusion criteria, have been used to find relevant studies for this meta-analysis (Figure 2 and Figure 3).

### 2.3. Data Extraction

Two independent reviewers screened the titles and abstracts of all the identified articles. Full-text articles of potentially eligible studies were retrieved and assessed for inclusion, with disagreements being resolved through discussion and, if necessary, by consulting a third reviewer. The same reviewers extracted the data using a standardised data extraction form. The following data were obtained: study characteristics (author, year, study design, and follow-up period), patient characteristics (sample size and gender), intervention details (type of interspinous spacer device used and the control used), outcomes (incidence and severity of ASD/ASDis). The primary outcome was the incidence of adjacent segment degeneration. Secondary outcomes included the range of motion (flexion/extension) at the adjacent segment, clinical and functional improvement based on the VAS and ODI score, adverse events related to the spacer devices, and presentation relative to the follow-up period.

### 2.4. Criteria for ASD 

Specific criteria were used to detect ASD, based on the literature search. This included:

- Disc height reduction of ≥50% [24].
- Spondylolisthesis of 4 mm [24].
- Angulation of >10 degrees on lateral flexion and extension radiographs [24,25].
- Grade IV or V Pfirrmann classification on magnetic resonance imaging (MRI)/computed tomography (CT) scans [26].
- Clinical findings at the adjacent segment—spinal stenosis, disc herniation, and mechanical back pain [27,28].

### 2.5. Risk of Bias Assessment

The risk of bias for individual studies was assessed using the Newcastle–Ottawa Scale (NOS), given these were all comparative retrospective cohort studies. Each study was evaluated for selection bias, performance bias, detection bias, attrition bias, and reporting bias, and a score of ≥6 suggested high-quality research. Discrepancies were also resolved by discussion or by consulting a third reviewer. Publication bias across all the studies was assessed using funnel plots and Egger’s test. A *p*-value of less than 0.05 was considered indicative of significant publication bias.

### 2.6. Statistical Analysis

The Review Manager 5.4 software was used for this meta-analysis. The primary measure of effect was the odds ratio (OR) for binary outcomes and the standard mean difference (SMD) for continuous outcomes, both with 95% confidence intervals (CIs). The χ^2^ test was employed with sensitivity analysis to assess the heterogeneity of the included studies. A *p*-value of ≥0.1 and an I^2^ value of ≤50% indicated no significant heterogeneity among the studies, thus warranting a fixed-effect model. Conversely, a *p*-value of less than 0.1 or an I^2^ value greater than 50% signified significant heterogeneity, in which case a random-effects model was applied. A sensitivity analysis was also performed for subgroup analyses that caused significant heterogeneity, thereby excluding outliers. 

To ensure that our meta-analysis was adequately powered to detect a statistically significant effect, we conducted a power analysis using R Studio. Specifically, we employed a simulation-based approach using the ‘metafor’ package to determine the minimum balanced sample size required to achieve a desired power of 90%. 

## 3. Results

### 3.1. Study Selection

A total of 1264 records were identified through database searching, and 4 additional records were identified through citation searching. After removing 11 duplicates, 1114 ineligible records, and 15 additional papers due to full-text availability, language (Chinese), date published, and article type, 124 records were screened. Of these, 51 records were excluded as these detoured from our research objectives, and a further 15 were not retrieved. Fifty-eight records were assessed for eligibility, with 67 excluded for various reasons, including 32 for lacking patient-reported outcomes, 16 for insufficient follow-up periods, and 4 for being systematic reviews and meta-analyses. A further three papers were excluded from the citations identified since two of these assessed the intervention of traditional lumbar fusion only, and one was in Chinese. Ultimately, seven studies were included in the qualitative and quantitative synthesis. The PRISMA flow diagram (Figure 1) provides a detailed overview of the study selection process.

### 3.2. Study Characteristics

The systematic review included a total of seven retrospective cohort studies that examined the effectiveness of IPDs in managing ASD following lumbar spinal fusion [16,17,29,30,31,32,33]. These studies varied in sample size, follow-up duration, and the specific interventions used.

The studies involved 546 participants, with a varied distribution of male and female patients. Each study was a retrospective cohort study, with the number of participants in individual studies ranging from 38 to 164. Different types of interspinous process devices were used across the studies, including Coflex, Wallis, DIAM, ROCKER, and SPIRE. The control groups in these studies generally consisted of patients who underwent PLIF or TLIF.

The primary outcomes measured were the incidence of ASD or adjacent segment disease. Secondary outcomes included various clinical metrics such as the VAS for lower back and leg pain, ODI, ROM, intraoperative blood loss, and operation time. The follow-up periods ranged from 2 to 3 years, as several studies did not include specific metrics prior to this period, although these were mentioned.

The post-operative complications reported in three of the studies included: intraspinal hematoma, subcutaneous incision infections, device migration, surgical site pain, and more severe issues such as cage migration, dural tears, and screw mispositioning [16,29,33]. There was not enough comparable quantitative data for specific post-operative complications between the studies. The reported complications are mentioned in Table 1.

### 3.3. Power Analysis

The power analysis revealed that a minimum of 32 participants per group is required to achieve 90%. However, it is important to note that some of the studies included in the meta-analysis had sample sizes smaller than 32 participants per group. This discrepancy suggests that these individual studies may have had insufficient power on their own to detect a significant effect. Despite this, the overall power of the meta-analysis was initially estimated at 0.982, indicating a very high likelihood of detecting a true effect if one exists, even with the inclusion of smaller studies. This high power reflects the cumulative effect of combining data across multiple studies, which increases the overall power of the meta-analysis beyond that of any single study.

### 3.4. Risk of Bias within Studies

All seven studies included in this systematic review were deemed of high quality, as they each received a score of 6 or higher on the NOS. Even though some studies scored slightly lower, with scores of 6, primarily due to issues in the comparability of cohorts and assessment of outcomes, these studies were still considered to provide valuable and reliable data for the meta-analysis. More details of the specific findings can be found in Table 2.

Overall, the assessment indicates a generally low risk of bias across the included studies, enhancing the credibility and reliability of the findings in this analysis.

### 3.5. Meta-Analysis Results 

#### 3.5.1. Incidence of ASD

**Figure 4:**
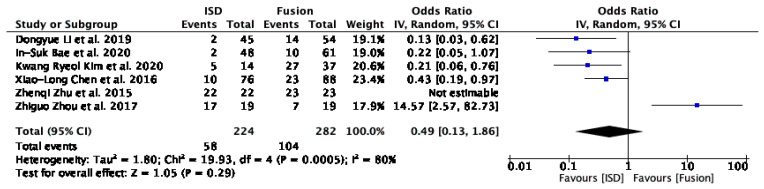
A forest plot showing the incidence of ASD [16,17,29,30,31,32].

The forest plot analysis for the incidence of adjacent segment degeneration (ASD) (Figure 4) included six studies: Li et al. [29], Bae et al. [16], Kim et al. [17], Chen et al. [31], Zhu et al. [30], and Zhou et al. [32]. The overall odds ratio (OR) was 0.49 (95% CI: 0.13 to 1.86), with a Z-value of 1.05 (*p* = 0.29), indicating no statistically significant difference in ASD incidence between IPD and fusion techniques. The analysis revealed substantial heterogeneity (I^2^ = 80%).

**Figure 5:**
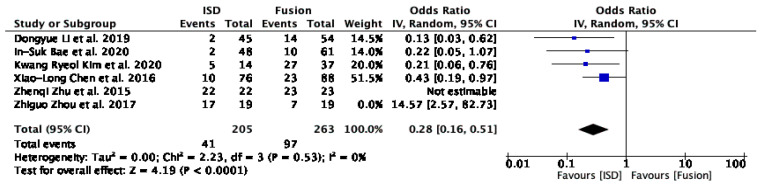
A forest plot for the incidence of ASD after removing outliers [16,17,29,30,31,32].

Excluding outliers like Zhou et al. [32] revealed a reduction in the incidence of ASD with the use of IPDs compared to fusion techniques, with an odds ratio (OR) of 0.28 (95% CI: 0.16 to 0.51 and *p* < 0.0001) (Figure 5).

#### 3.5.2. ODI Score

**Figure 6:**
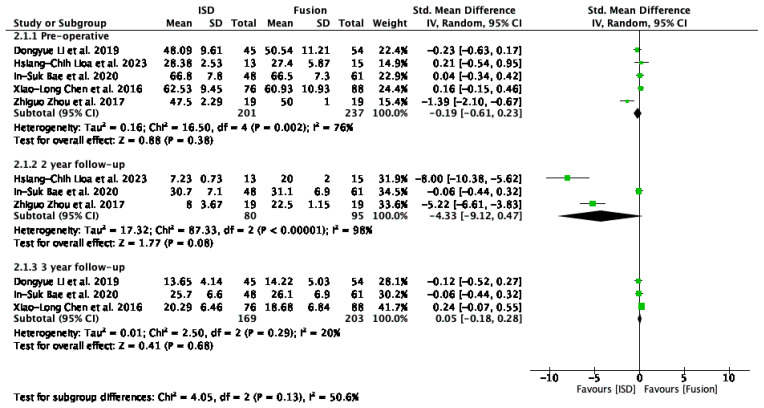
A forest plot showing the ODI score [16,29,31,32,33].

The analysis of ODI (Figure 6) scores included studies by Li et al. [29], Liao et al. [33], Bae et al. [16], Chen et al. [31], and Zhou et al. [32], evaluated pre-operatively, at the 2-year follow-up, and at the 3-year follow-up. Pre-operatively, the overall standardised mean difference (SMD) was −0.19 (95% CI: −0.61 to 0.23); at the 2-year follow-up it was −4.33 (95% CI: −9.12 to 0.47), and at the 3-year follow-up it was 0.05 (95% CI: −0.18 to 0.28). None of these results were statistically significant. Heterogeneity was high pre-operatively and at a 2-year follow-up. 

**Figure 7:**
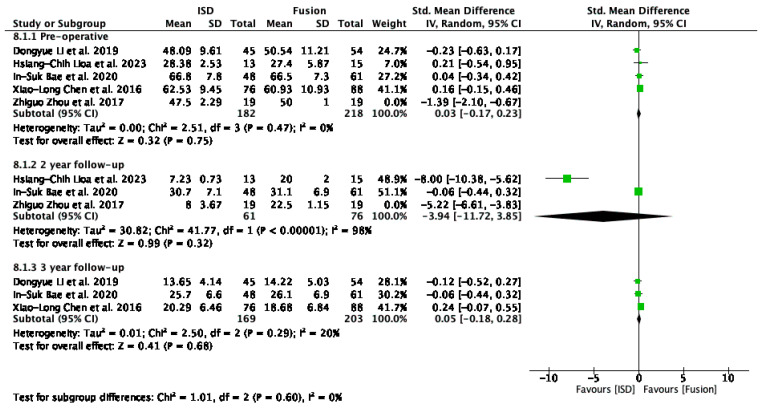
A forest plot for ODI scores after removing outliers [16,29,31,32,33].

The removal of outliers like Zhou et al. [32] revealed no significant difference in functional disability outcomes between interspinous process devices (IPDs) and fusion techniques pre-operatively (SMD = 0.03, 95% CI: −0.17 to 0.23) and at the 3-year follow-up (SMD = 0.05, 95% CI: −0.18 to 0.28) (Figure 7). 

#### 3.5.3. Range of Motion

**Figure 8:**
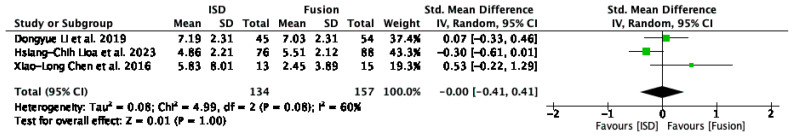
A forest plot showing the range of motion [29,31,33].

The forest plot for the range of motion (ROM) (Figure 8) included three studies: Li et al. [29], Liao et al. [33], and Chen et al. [31]. The overall SMD was −0.00 (95% CI: −0.41 to 0.41), with a Z-value of 0.01 (*p* = 1.00), indicating no statistically significant difference in ROM between IPD and fusion techniques. Heterogeneity was moderate (I^2^ = 60%).

#### 3.5.4. VAS Lower Back Pain Score

**Figure 9:**
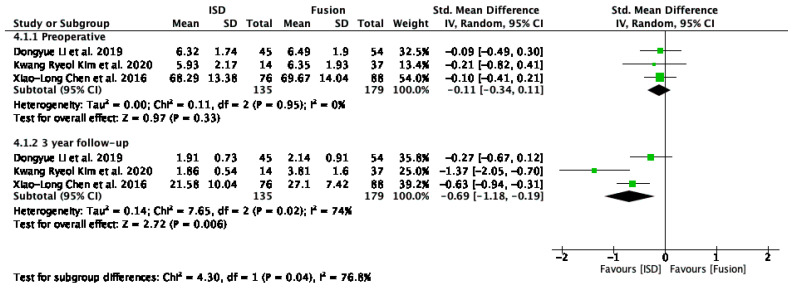
A forest plot showing the VAS lower back pain score [17,29,31].

The VAS lower back pain scores analysis compared IPD to fusion techniques at pre-operative and 3-year follow-up periods (Figure 9). The studies included were by Li et al. [29], Kim et al. [17], and Chen et al. [31]. Pre-operatively, the overall SMD was −0.11 (95% CI: −0.34 to 0.11), and was not statistically significant. At the 3-year follow-up, the overall SMD was −0.69 (95% CI: −1.18 to −0.19), indicating a statistically significant reduction in VAS lower back pain scores, favouring IPD. Heterogeneity was substantial at a 3-year follow-up (I^2^ = 74%).

#### 3.5.5. VAS Leg Pain Score

**Figure 10:**
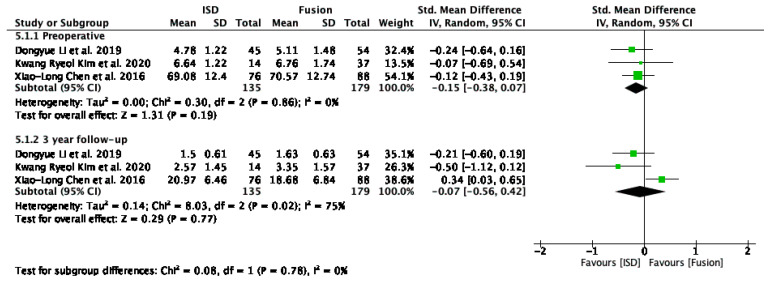
A forest plot showing VAS leg pain scores [17,29,31].

The forest plot for VAS leg pain scores (Figure 10) included studies by Li et al. [29], Kim et al. [17], and Chen et al. [31], analysed pre-operatively and at a 3-year follow-up. Pre-operatively, the overall SMD was −0.15 (95% CI: −0.38 to 0.07), and at the 3-year follow-up, it was −0.07 (95% CI: −0.56 to 0.42), with both not statistically significant. Heterogeneity was low pre-operatively (I^2^ = 0%) but high at the 3-year follow-up (I^2^ = 75%).

**Figure 11:**
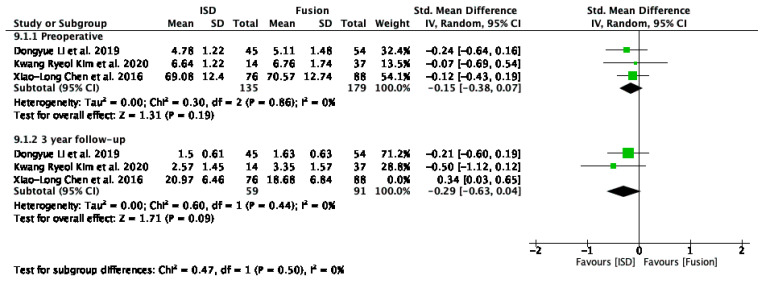
A forest plot for VAS leg pain scores after removing outliers [17,29,31].

Excluding the outlier study by Chen et al. [31] in the analysis of VAS scores for leg pain (Figure 11), the results showed consistent improvements in leg pain scores with IPDs compared to fusion techniques, with an SMD of −0.29 (95% CI: −0.63 to 0.04).

#### 3.5.6. Intraoperative Blood Loss

**Figure 12:**
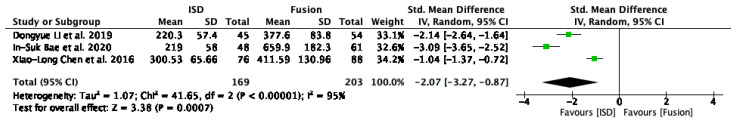
A forest plot showing intraoperative blood loss [16,29,31].

The forest plot for intraoperative blood loss (Figure 12) included studies by Li et al. [29], Bae et al. [16], and Chen et al. [31]. The overall SMD was −2.07 (95% CI: −3.27 to −0.87), with a Z-value of 3.38 (*p* = 0.0007), indicating a statistically significant reduction in intraoperative blood loss favouring IPD. Heterogeneity was substantial (I^2^ = 95%).

#### 3.5.7. Operation Time

**Figure 13:**
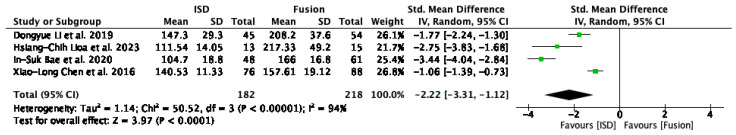
A forest plot showing operation time [16,29,31,33].

The analysis of operation time (minutes) (Figure 13) compared IPD techniques to fusion techniques across four studies: Li et al. [29], Liao et al. [33], Bae et al. [16], and Chen et al. [31]. The overall SMD was −2.22 (95% CI: −3.31 to −1.12), with a Z-value of 3.97 (*p* < 0.0001), indicating a statistically significant reduction in operation time favouring IPD. Heterogeneity was substantial (I^2^ = 94%).

#### 3.5.8. Publication Bias

**Figure 14:**
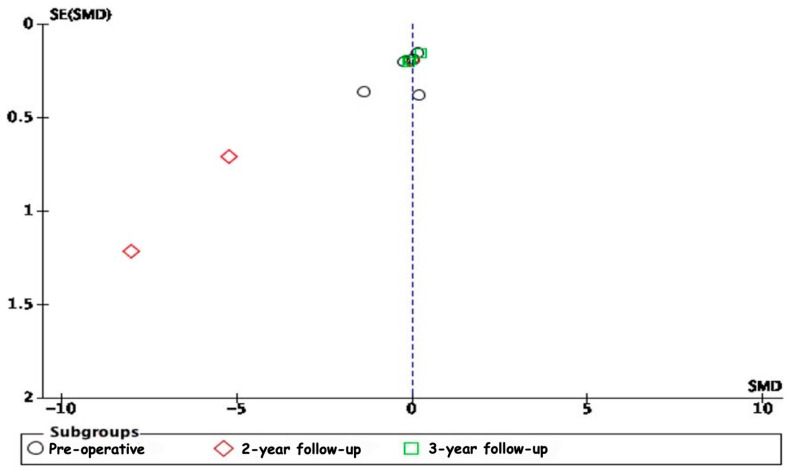
A funnel plot for the ODI scores.

**Figure 15:**
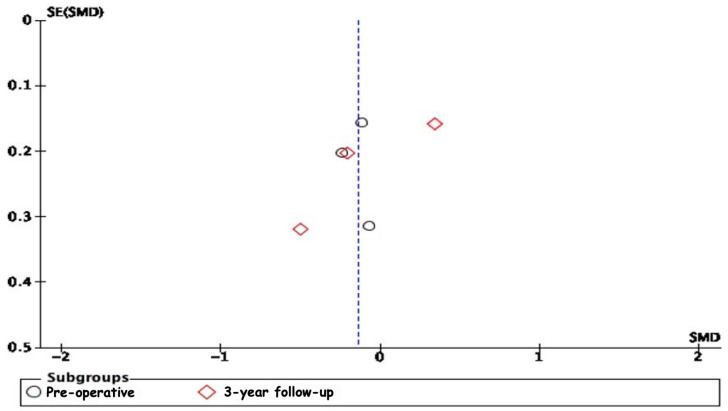
A funnel plot for VAS leg pain scores.

**Figure 16:**
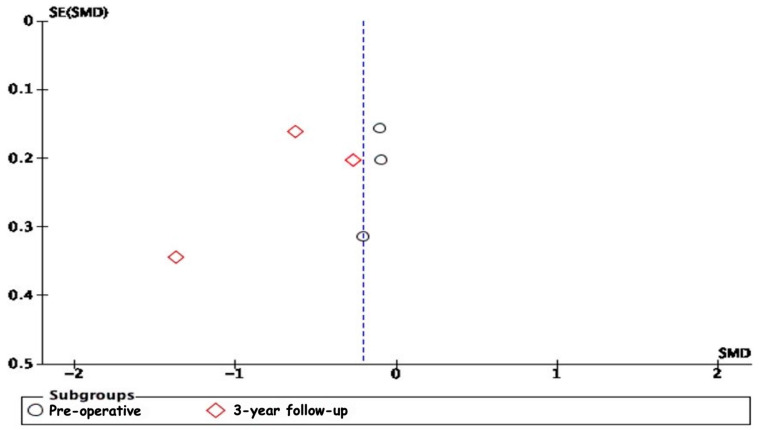
A funnel plot for VAS lower back pain scores.

The analysis of publication bias through funnel plots revealed potential biases in several of the meta-analyses conducted. The asymmetry observed in the funnel plots, particularly in the 2-year follow-up subgroup for ODI scores and the 3-year follow-up subgroups for VAS leg pain scores and VAS lower back pain scores, suggests the presence of publication bias or other small-study effects (Figure 14, Figure 15 and Figure 16). Specifically, smaller studies showing larger effects may disproportionately influence the overall results, indicating that the positive findings might be overestimated due to the selective publication of studies with significant results. The moderate to high heterogeneity observed in these analyses further complicates the interpretation, as it underscores a variability in study outcomes likely stemming from differences in study design, population characteristics, and intervention methods. 

### 3.6. Sensitivity Analysis

A sensitivity analysis was conducted to address the significant heterogeneity by systematically excluding outliers. For the ODI scores, removing Zhou et al. [32] reduced heterogeneity to I^2^ = 37.3%. Similarly, excluding Zhou et al. for the incidence of adjacent segment degeneration (ASD) eliminated heterogeneity entirely (I^2^ = 0%). In the VAS leg pain score analysis, removing Chen et al. [31] also eliminated heterogeneity (I^2^ = 0%). However, for intraoperative blood loss, operation time, ROM, and VAS lower back pain, excluding the outliers did not significantly reduce heterogeneity, which remained high (I^2^ > 50%). These findings indicate that while heterogeneity was reduced considerably for ODI, ASD, and VAS leg pain scores by excluding specific studies, other measures exhibited substantial variability.

## 4. Discussion

The primary aim of this systematic review and meta-analysis was to evaluate the effectiveness of IPDs in managing ASD following lumbar spinal fusion. The results of our analysis indicate several important findings that contribute to understanding the comparative benefits of IPDs versus traditional fusion techniques.

Our meta-analysis revealed a reduction in the incidence of ASD with the use of IPDs compared to fusion techniques, with an OR of 0.28 (95% CI: 0.16 to 0.51, *p* < 0.0001) (Figure 5). This suggests that IPDs for ASD may provide a protective effect against the development of new-onset ASD [14]. Eliminating heterogeneity (I^2^ = 0%) after excluding outliers like Zhou et al. [32] further supports this finding. However, the challenges in quantifying ASD in certain studies, such as that of Liao et al. [33], where clinical and radiological evidence confirmed its presence post-operatively, and Zhu et al. [30], who reported a 100% incidence rate for both IPD and fusion groups, highlight the need for standardised criteria in future research.

### 4.1. Functional Outcomes

The analysis of ODI scores revealed no significant difference in functional disability outcomes between interspinous process devices and fusion techniques preoperatively (SMD = 0.03, 95% CI: −0.17 to 0.23) and at the 3-year follow-up (SMD = 0.05, 95% CI: −0.18 to 0.28) (Figure 7). However, a non-significant trend towards improved outcomes with IPDs was noted at the 2-year follow-up (SMD = −3.94, 95% CI: −11.72 to 3.85) despite high heterogeneity (I^2^ = 98%). Excluding the study by Zhou et al. [32] significantly reduced heterogeneity overall (I^2^ = 0%), underscoring the importance of methodological consistency. These findings indicate that IPDs are as effective as fusion techniques in managing functional disability in patients with ASD following lumbar spinal fusion. The comparable outcomes support the use of IPDs as a viable alternative to fusion but emphasise the need for standardised reporting to improve the reliability of future research in this area.

### 4.2. Preservation of Range of Motion

Our findings indicated that IPDs were associated with a similar preservation of ROM compared to fusion techniques, with an SMD of −0.00 (95% CI: −0.41 to 0.41, *p* = 1.00). The studies included in this analysis varied in their methods of measuring ROM. For instance, Li et al. [29] used general adjacent segment mobility (GASM) to calculate ROM at L2-L4, while Chen et al. [31] and Liao et al. [33] presented ROM results without specifying the spinal levels.

As demonstrated by Cao et al. [34], topping off with an IPD is expected to reduce ROM at the adjacent segment compared to fusion, with the aim of preventing hypermobility and subsequent disc degeneration. Our study’s effect on ROM warrants further analysis of the effect of ROM preservation and its impact on the development of ASD.

### 4.3. Pain Relief

The VAS scores for lower back pain showed a significant reduction in pain levels at the 3-year follow-up for patients treated with IPDs compared to fusion techniques. The SMD for VAS scores was −0.69 (95% CI: −1.18 to −0.19), indicating that IPDs provide superior pain relief over the long term. The high heterogeneity observed in the follow-up period (I^2^ = 74%) suggests that differences in patient populations, surgical techniques, and follow-up durations across studies may have contributed to variability in pain outcomes. However, the overall reduction in VAS scores highlights the potential of IPDs to provide sustained pain relief by preserving spinal motion and reducing mechanical stress.

Excluding the study by Chen et al. [31] in the analysis of VAS scores for leg pain (Figure 11) significantly reduced heterogeneity for the 3-year follow-up (I^2^ = 0%), suggesting that this study’s results were an outlier. The remaining studies showed consistent improvements in leg pain scores with IPDs compared to fusion techniques, with an SMD of −0.29 (95% CI: −0.63 to 0.04). This reduction in leg pain might be attributed to the ability of IPDs to provide indirect neural decompression while preserving segmental motion, thereby preventing the exacerbation of leg pain often associated with traditional fusion techniques [35].

### 4.4. Intraoperative Benefits

Interspinous process devices offer significant advantages over fusion techniques in terms of operative time and blood loss. Our analysis demonstrated that IPDs were associated with significantly lower intraoperative blood loss, with an SMD of −2.07 (95% CI: −3.27 to −0.87), and shorter operation times, with an SMD of −2.22 (95% CI: −3.31 to −1.12). These benefits are likely due to the minimally invasive nature of IPD procedures, which involve less extensive surgical dissection and fewer surgical steps. This reduction in operative time and blood loss can decrease the risk of intraoperative complications and anaesthesia-related adverse events, making IPDs a viable alternative over traditional fusion techniques, particularly for patients at high surgical risk who might benefit from reduced perioperative morbidity and faster recovery.

### 4.5. Safety and Emerging Evidence

The safety of top-off surgery with IPDs for ASD has been established as a feasible and safe technique. Studies by Fuster et al. (2022) [36] and Nachanakian et al. (2013) [37] confirm this finding, highlighting the potential of IPDs to serve as a dynamic fixation method to prevent ASD without significantly compromising safety [36,37]. Recently, a study assessing the usage of percutaneous salvage therapy for ASD demonstrated results in concordance with those presented in our study. This use of IPDs might be beneficial for patients who are unwilling to undergo or are unfit for corrective deformity surgery [38]. However, its benefits in comparison to a traditional extension of fusion have yet to be proven, and its superiority has not been established. Controversy remains regarding the use of IPDs, due to study methodological inconsistencies and high variability in interspinous devices’ design and purpose.

### 4.6. Considerations for Future Research

The use of IPDs might be sufficient to maintain sagittal balance, as suggested by Schulte et al. (2011) [39], in patients undergoing surgery for adjacent level disease, thus precluding the need for more invasive techniques aimed at restoring sagittal alignment [39]. However, downsides to IPDs have been reported, including higher reoperation rates compared to laminectomy alone. This was observed by Meyer et al. (2018) [40], although this finding might not be directly transferable to ASD treatment and was not observed in our study.

### 4.7. Limitations

This present study was not registered in PROSPERO, as we became aware of the registration process late in the study. While we did not register our meta-analysis with PROSPERO, we documented our study protocol thoroughly, finalising it before data extraction began to ensure that our methodology was pre-specified and transparent. We have adhered to the PRISMA guidelines, providing a comprehensive and transparent account of our methodology.

The included studies featured a diverse patient population with mean ages ranging from 40 to 67.1 years in the fusion groups and from 44.5 to 68.16 years in the IPD groups. Despite this range, the population that might benefit the most from this minimally invasive technique—specifically, older patients—was not sufficiently represented. This demographic is particularly relevant as older patients, who often present with more complex comorbidities, could potentially gain more from the reduced invasiveness and quicker recovery times associated with IPDs [41,42].

Moreover, the duration of follow-up in the studies, which ranged from 2 to 3 years, is relatively short for assessing long-term outcomes and the true incidence of ASD [43]. Given that ASD can manifest several years post-fusion, follow-up periods exceeding five years are necessary to provide a more accurate evaluation of the sustained efficacy and safety of IPDs. This extended observation is crucial to determine whether IPDs can effectively delay or prevent the onset of ASD in the long term.

Another critical factor that is inconsistently addressed in the studies is sagittal balance. The studies reviewed did not consistently assess pre- and post-operative sagittal alignment, despite its importance for improved long-term outcomes [44]. The preservation or restoration of sagittal balance is now recognised as a critical component in fusion surgeries, influencing the time to revision surgery and overall patient outcomes [45]. Studies often lacked detailed assessments of sagittal balance, focusing instead on radiographic parameters like intervertebral mobility and disc height. These parameters provide limited insight into the overall biomechanical impact of the surgery. Future research should prioritise a comprehensive evaluation of sagittal balance both pre- and post-operatively. Understanding how IPDs influence sagittal balance can provide valuable insights into their effectiveness in preventing ASD and improving long-term patient outcomes.

Furthermore, the specifics of fusion levels varied among the included studies, with some focusing on single-level fusions, while others included multi-level fusions. For instance, Li et al. [29] examined L3-5 fusion for the PLIF group and L4-5 PLIF combined with L3-4 Coflex for the topping-off group. However, other studies did not clearly detail the fusion levels, as seen in the works by Zhou et al. [32] and Bae et al. [16]. This variation underscores the necessity for standardised reporting in future studies to ensure comparability and clarity.

In summary, the data from our study must be interpreted with caution due to the short follow-up duration and inconsistencies in reporting baseline fusion levels and sagittal alignment. These limitations highlight the need for further studies to focus on the clinical effects of sagittal alignment and the use of interspinous process devices (IPDs). Future research should aim for more rigorous and long-term assessments to provide a comprehensive understanding of the outcomes associated with IPD use, particularly in relation to preserving spinal alignment and preventing ASD.

Additionally, there is a need to assess the impact of patient-specific parameters and pathology on the efficacy of IPDs. Understanding the relationship between these factors and sagittal alignment is crucial for tailoring treatments to individual patients.

## 5. Conclusions

Our findings suggest that the judicious use of IPDs might benefit a subset of patients, particularly those who are not suitable candidates for major corrective surgery. Such insights underscore the potential for IPDs to offer a valuable alternative treatment option, warranting further investigation.

## Figures and Tables

**Table 1:** Characteristics of included literature.

Author	Study Design	Number of Patients (Male (M)/Female (F))	Intervention Used	Control	Incidence of ASD/ASDis	Outcome Measures	Follow-Up	Post-Operative Complications
Dongyue Li et al. 2019 [29]	Retrospective cohort study	99 (46 M/53 F)	PLIF + Coflex	PLIF	ASD	VAS, ODI, ROM, Lumbar MRI	3 years	Intraspinal haematoma, subcutaneous incision infection
Zhenqi Zhu et al. 2015 [30]	Retrospective cohort study	45 (25 M/20 F)	PLIF + Wallis	PLIF	ASD	VAS, JOA	1 year	NA
Xiao-Long Chen et al. 2016 [31]	Retrospective cohort study	164 (92 M/72 F)	PLIF + Coflex	PLIF	ASD	VAS, ODI, ROM, FW, LL, SS, Lumbar MRI, CT	3 years	NA
Kwang Ryeol Kim et al. 2020 [17]	Retrospective cohort study	51 (21 M/30 F)	PLIF + DIAM	PLIF	ASD	VAS, RMDQ, ROM, MRI	2 years	NA
Zhiguo Zhou et al. 2017 [32]	Retrospective cohort study	38 (21 M/17 F)	Discectomy + Wallis	Discectomy	ASD	VAS, ODI. MRI	2 years	NA
Hsiang-Chih Lioa et al. 2023 [33]	Retrospective cohort study	40 (12 M/28 F)	TLIF + ROCKER (Paonan, Taipei, Taiwan)	TLIF	ASD	VAS, ODI, ROM	2 years	Infection, device breakage, pain at surgical site
In-Suk Bae et al. 2020 [16]	Retrospective cohort study	109 (62 M/47 F)	PLIF + SPIRE (Medtronic Sofamor Danek, Dublin, Ireland)	PLIF	ASD	VAS, ODI, CT	3 years	Cage migration, dural tears, revision surgery, screw mispositioning, rod breakage

Abbreviations: DIAM^®^ (Device for Intervertebral Assisted Motion, Medtronic Ltd., Dublin, Ireland), Coflex™ device (Paradigm Spine, LCC, New York, NY), Wallis dynamic stabilization system (Abbott Spine, Bordeaux, France), PLIF—posterior lumbar interbody fusion, TLIF—transforaminal lumbar interbody fusion, JOA—Japanese Orthopaedic Association score, ROM—range of motion, LL—lumbar lordosis, SS—sacral slope, RMDQ—Roland–Morris Disability Questionnaire, FW—foraminal width.

**Table 2:** Results of the quality assessment using the Newcastle–Ottawa Scale for cohort studies.

Study	Representativeness of the Exposed Cohort	Selection of the Non-Exposed Cohort	Ascertainment of Exposure	Demonstration That Outcome of Interest Was Not Present at Start of Study	Comparability of Cohorts on the Basis of the Design or Analysis	Assessment of Outcome	Follow-Up Long Enough for Outcomes to Occur	Adequacy of Follow-Up of Cohorts	Quality Score
Dongyue Li et al. 2019 [29]	1	1	1	1	1	1	1	1	8
Zhenqi Zhu et al. 2015 [30]	1	1	1	1	0	0	1	1	6
Xiao-Long Chen et al. 2016 [31]	1	0	1	1	1	1	1	1	7
Kwang Ryeol Kim et al. 2020 [17]	1	1	1	1	0	0	1	1	6
Zhiguo Zhou et al. 2017 [32]	1	1	1	1	0	0	1	1	6
Hsiang-Chih Lioa et al. 2023 [33]	1	1	1	1	1	1	1	1	8
In-Suk Bae et al. 2020 [16]	1	1	1	1	0	0	1	1	6

## Data Availability

The original contributions presented in the study are included in the article; further inquiries can be directed to the corresponding authors.

## Abbreviations

ASD	Adjacent Segment Degeneration
ASDis	Adjacent Segment Disease
IPD	Interspinous Process Devices
PRISMA	Preferred Reporting Items for Systematic Reviews and Meta-Analyses
MeSH	Medical Subject Headings
PICO	Patient, Intervention, Comparison, and Outcome
VAS	Visual Analog Scale
ODI	Oswestry Disability Index
RCT	Randomised Controlled Trial
MRI	Magnetic Resonance Imaging
CT	Computed Tomography
PLIF	Posterior Lumbar Interbody Fusion
ROM	Range of Motion
JOA	Japanese Orthopaedic Association
FW	foraminal width
RMDQ	Roland–Morris Disability Questionnaire
LL	Lumbar Lordosis
SS	Sacral Slope
NOS	Newcastle–Ottawa Scale
OR	Odds Ratio
CI	Confidence Interval
TLIF	Transforaminal Lumbar Interbody Fusion
SMD	Standard Median Deviation
GASM	General Adjacent Segment Mobility
